# Association between Socioeconomic Factors, Food Insecurity, and Dietary Patterns of Adolescents: A Latent Class Analysis

**DOI:** 10.3390/nu15204344

**Published:** 2023-10-12

**Authors:** Vanessa Barbosa Facina, Rosemary da Rocha Fonseca, Maria Ester Pereira da Conceição-Machado, Rita de Cássia Ribeiro-Silva, Sandra Maria Chaves dos Santos, Mônica Leila Portela de Santana

**Affiliations:** 1 Postgraduate Program in Food, Nutrition and Health, School of Nutrition, Federal University of Bahia, Salvador 40170-110, Brazil; 2 Health Sciences Center, Federal University of Recôncavo da Bahia, Santo Antonio de Jesus 44574-490, Brazil; 3 Nutrition Science Department, School of Nutrition, Federal University of Bahia, Salvador 40170-110, Brazil

**Keywords:** dietary patterns, latent class analysis, socioeconomic status, adolescents, food insecurity

## Abstract

Socioeconomic factors and food insecurity play a fundamental role in the food choices of adolescents, and in addition to influencing access to food, they also have significant effects on dietary patterns. The objectives of this study were to identify the dietary patterns of adolescents through the application of latent class analysis and to evaluate their association with socioeconomic variables and food insecurity. This cross-sectional study was conducted with adolescents aged between 11 and 17 years from public schools. Latent class analysis was used to identify the dietary patterns. Associations between socioeconomic factors, food insecurity and dietary patterns were assessed using multinomial logistic regression (odds ratio (OR); 95% confidence interval (CI)). Among the 1215 participants in the study, four dietary patterns were identified: “Mixed”, “Low consumption”, “Prudent” and “Diverse”. A “Diverse” dietary pattern was associated with a lower economic stratum (OR:2.02; CI:1.26–3.24). There was no association between food insecurity and identified dietary patterns. These results highlight the importance of promoting healthy eating in this age group at all socioeconomic levels, especially the lowest level.

## 1. Introduction

Adolescence is a period of physical, psychological, and emotional change associated with great vulnerability to external factors (environmental, cultural, social, economic, and media) that interact directly and indirectly with various behaviors that are perpetuated throughout life, such as eating behaviors [1,2]. Socioeconomic factors and food insecurity (FI) play a fundamental role in adolescents’ food choices as they can influence access to food and have significant effects on dietary patterns [2,3]. In addition, economic deprivation and social problems are associated with the adoption of less healthy eating practices, predicting an increased risk of developing diseases related to diet and obesity [4,5,6,7].

To analyze eating practices, it is important to consider that food intake comprises a variety of foods with complex combinations of nutrients and other food components [8]. Thus, dietary pattern analysis has been increasingly used because it is considered a more realistic representation of eating practices [5,8,9,10,11]. Among adolescents, the most frequent dietary patterns have been those called “unhealthy or junk food or Western”, “healthy”, “mixed or traditional diet”, and “snack” [6].

The main methods used to assess dietary patterns (DP) are factor analysis (principal component analysis or common factor analysis) and cluster analysis [6,9]. However, other methods have emerged, such as latent class analysis (LCA) which, unlike factor analysis, can classify individuals into mutually exclusive dietary patterns, and, unlike cluster analysis, makes it possible to quantify the uncertainty in class membership and assess the model fit quality [8,11,12,13]. LCA aims to identify subgroups of people who share common characteristics and allows the establishment of the optimal number of dietary patterns or to estimate the probability that each individual belongs to each of these patterns [13,14].

Widely used in social sciences, LCA has proven to be a useful approach to classify individuals into classes based on the similarity of the behavior that is intended to be studied [14]. Thorne-Lyman et al. (2020) revealed a great diversity of eating behaviors by identifying five dietary patterns among adolescents in rural Bangladesh [11]. Among Portuguese adolescents, two dietary patterns were identified and evidenced the high adherence to the “unhealthy” eating pattern, characterized especially by the higher consumption of sugar-sweetened beverages and ultra-processed foods, in addition to lower consumption of fruits, vegetables, and legumes [12]. A study of Brazilian adolescents using LCA identified four dietary patterns that highlighted the high consumption of unhealthy, non-regional foods and a certain dietary monotony [15].

Because it is a precise and sophisticated form of grouping, LCA can be used to assess dietary patterns, helping to better understand the eating practices adopted in adolescence [16]. The present study aimed to identify and describe food consumption patterns among adolescents through the application of LCA and to evaluate their association with socioeconomic variables and food insecurity. It is hypothesized that low socioeconomic status and/or household food insecurity predict greater adherence to a dietary pattern that includes foods such as fast food, sweets, sugars, and sugar-sweetened beverages.

## 2. Materials and Methods

### 2.1. Study Design and Population

This was a population-based cross-sectional study conducted with male and female adolescents aged between 11 and 17 years who enrolled in 2009 in the public school system of Salvador, Bahia, Brazil. It is part of a larger study entitled “Fatores psicossociais como elementos que repercutem nas condições de saúde, nutrição e desenvolvimento cognitivo de estudantes do ensino fundamental das escolas públicas de Salvador/BA” (Psychosocial factors as elements that impact the health, nutrition, and cognitive development conditions of elementary school students from public schools in Salvador/BA), whose sample calculation was carried out through a complex cluster approach in two stages: the first represented by schools, and the second by the classes, having 1496 adolescents as the unit of analysis [17]. Pregnant and/or lactating girls and students with physical problems that prevented anthropometric assessment were not eligible to participate in the study.

For the present study, a sample of adolescents whose parents or guardians answered the Brazilian Food Insecurity Scale (BFIS) was used because it is an important variable for tracing the socioeconomic characteristics of adolescents, totaling 1215 participants.

### 2.2. Food Intake

Data on adolescents’ food intake were self-reported using a semi-quantitative food frequency questionnaire (FFQ) with 97 food items, which was validated by Mascarenhas et al. (2014). This instrument contains the frequency of consumption of food items structured as follows: never/rarely; one–three times a month, once a week, two–four times a week; four times a week, or more often. The number of times food was consumed per day was also obtained [18].

To calculate the daily intake (in grams and/or milliliters) of foods/preparations in the FFQ, the portion sizes of each food/preparation were standardized using cooking measurement tables as references [19,20]. The amount of daily intake of each food/preparation was obtained from the frequency of intake indicated by the adolescents. Processed foods and/or preparations that were not included in the cooking measurement tables were researched on the internet, directly on the manufacturer’s and/or recipe websites.

After conversion into grams, the food items in the FFQ were clustered by adding the total number of grams for each item to form food groups. To create these food clusters, the extent and purpose of the processing of the food items as well as the similarity of the nutrient content were considered. Recent studies have employed this form of food classification, in which food processing is defined as “all methods and techniques used by the food, beverage, and associated industries to transform whole fresh foods into food products” [21]. Sixteen food groups were identified in this study (Table 1).

### 2.3. Transformation of Variables and Creation of Dietary Patterns

LCA uses categorical variables as inputs for the analyses, and based on the total consumption (in grams) of each food group, variables that reflected the relative consumption of each food group in the population were created. Thus, the food groups were categorized into tertiles, quintiles, and percentiles, with the generation of 3 models to be analyzed. The first model classified consumption as <2nd tertile (0) or ≥2nd tertile (1). The second model was classified as 4th quintile (0) or ≥4th quintile (1) and finally, the third model classified consumption as below P75 (0) or equal to or above P75 (1). Cutoff points, in grams of 75th percentile intake of food groups according to dietary pattern are available in Table S1. Each model gave rise to two to five latent classes (dietary patterns), which were evaluated for statistical quality. After defining the best model for representing dietary patterns, the names of the latent classes (dietary patterns) were defined according to the conditional probabilities of food intake [11,12,14].

### 2.4. Other Variables 

The age of the adolescents was calculated in years based on the difference between the date of the interview and the date of birth. For the analysis, it was categorized as under 14 years of age and 14 years of age or over. Sex was self-reported and categorized as male or female.

Information about the economic stratum and food security was provided by parents or guardians. To classify the economic stratum of the households, the Brazilian Criteria of Economic Classification (BCEC) were used to evaluate the level of education of the reference person in the household and the possession of items of domestic comfort, such as the number of bathrooms, monthly paid maids in the household, and durable goods [22]. A score was assigned for each item, and the total number of points was added at the end. Then, classification into strata (A1, A2, B1, B2, C1, C2, D, and E) was performed, with the lowest stratum being “E”. For this study, due to the non-existence of households in strata A1, A2, B1, and B2, this variable was re-categorized into two strata: “Stratum C and D” (the union of strata C1, C2, and D) and “Stratum E”. This stratum, “E”, represents a lower economic situation of households when compared to stratum “C and D”.

The Brazilian Scale of Food Insecurity (BFIS) was used to measure families’ perceptions regarding access to food by directly investigating their food security situation. It has been adapted and validated for the Brazilian population [23]. This instrument comprises questions related to respondents’ perception of the food situation at home in the last three months, and its dichotomous scoring assigns 1 (one) point to “yes” answers and 0 (zero) to “no”. According to total points, each household can be classified into food security, mild food insecurity, moderate food insecurity, and severe food insecurity [23].

### 2.5. Ethical Aspects

The study protocol was approved by the Research Ethics Committee of the Institute for Collective Health at the Federal University of Bahia (Instituto de Saúde Coletiva da Universidade Federal da Bahia) (protocol number 002/08). After being informed of the study objectives, parents or guardians of the adolescents signed an Informed Consent Form. Illiterate parents consented to using their fingerprints.

### 2.6. Statistical Analysis

The classification of individuals according to their dietary patterns was based on the probabilities of class membership (unconditional probability or average posterior probability), considering that each individual belongs to only one latent class, and the probability of item response (conditional probability), that is, the probability that the individual reports each item that constitutes the latent class [14,24].

The decision on the number of classes (dietary patterns) in the model is a central element of LCA and is commonly based on statistical measures of base model fit that consider the statistical quality of the model (parsimony) and the interpretability of the classes, which is based on the underlying theory of the latent construct that is being identified (dietary pattern) [14]. 

The models were evaluated for fit using raw and adjusted Bayesian information criteria (BIC and aBIC, respectively), likelihood ratio tests LMR-LRT (Lo–Mendell–Rubin Likelihood Ratio Test), and BS-LRT (Bootstrap Likelihood Ratio Test). The information criteria consider the complexity of the model, as they favor a more parsimonious model, and consider the model with the lowest value as the best fit. The LMR and Bootstrap tests are assumed to be the null hypothesis (H0) that the model with the number of classes K (−1) best fits the data, while the working hypothesis (H1) is that the model with K classes best fits the data; thus, *p* < 0.05 rejects the null hypothesis, thus considering that the model with K classes has the best fit [24].

The assumption for using LCA is conditional independence, that is, the observed variables should not be highly correlated within the identified classes. To evaluate this assumption, z-score standardized residuals from bivariate analyses were used, which were considered significant when greater than 1.96~2.00 [14,24]. The LCA models were implemented using the Mplus software version 5.0.

Multinomial logistic regression and 95% confidence intervals (95% CIs) were used to assess the association between socioeconomic factors and food insecurity (independent variables), and dietary patterns (dependent variable). The dietary pattern used as a reference was “Mixed” [12]. The significance level adopted for all analyses was set at 5% (*p* < 0.05). Considering the study design for complex sampling, the *svy* command was used. Statistical software STATA version 15.1 (Stata Corporation, College Station, TX, USA) was used.

## 3. Results

### 3.1. Dietary Patterns

**Figure 1:**
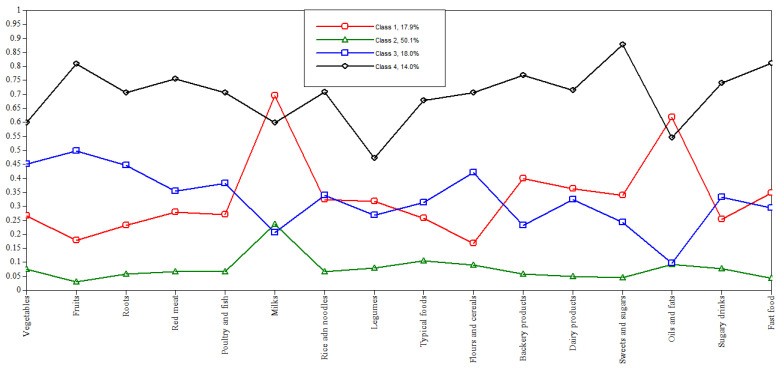
Chart of conditional probabilities of model 3 for four latent classes, 16 food groups.

When adjusting for the latent class models, the third model with four (4) latent class was chosen (BIC = 19,246.623; aBIC = 19,033.804; LMR-LRT = −9430.225; *p* < 0.5203; BS-LRT = −9430.225; *p* < 0.0000) to represent the dietary patterns of adolescents in this study (Table 2). Thus, upon careful examination of the analyzed criteria, the third model with four dietary patterns proved to be more parsimonious and allowed for better interpretability when identifying food intake patterns (Figure 1). Furthermore, the conditional independence assumption analysis was not violated, with 1.25% bivariate standardized residual z-scores in excess of 1.96~2.00 (6 of 480 bivariate residuals).

The first dietary pattern, called “Mixed”, was found in 17.9% (*n* = 192) of the adolescents, who had a greater tendency to consume milk, oils and fats, bakery and dairy products, fast food, sweets and sugar, rice and noodles, and legumes, and were less like to consume sugar-sweetened beverages, roots and tubers, fruits, flour, and cereals. DP2 was labeled “Low consumption”, as adolescents (50.1%; *n* = 644) who adhered to this pattern had lower consumption of practically all food groups in the ≥P75 percentile level, mainly dairy products, typical foods, oils and fats, flours and cereals. DP3 was characterized by a higher tendency to consume fruits, vegetables, roots and tubers, flour and cereals, poultry, and fish, and a lower tendency to consume oils and fats, bakery products, dairy products, sweets, and sugar, and was labeled “Prudent”, with 18.0% (*n* = 209) of the participants adhering to it. Participants who adhered to DP4 (14.0%; *n* = 170), called “Diverse”, were high consumers of virtually all food groups in the ≥P75 percentile level, especially sweets and sugar, fast food, fruits, bakery products, red meat, and sugar-sweetened beverages (Table 3).

### 3.2. Prevalence of Dietary Patterns and Relations with Socioeconomic Factors and Food Insecurity

Of the 1215 adolescents who participated in this study, the majority were female (57.8%), aged 14 years or older (56.5%), and living in households with some degree of food insecurity (66.3%). The prevalence of the “Mixed” DP was higher among adolescents from the “C and D” economic stratum, and the “Diverse” DP among adolescents from the “E” stratum (*p* = 0.022). No statistically significant differences were found between the prevalence of dietary patterns and sex, age, or household food insecurity (Table 4).

When analyzing the relationships between economic strata and dietary patterns, an association was found between the “Diverse” dietary pattern and the “E” economic stratum (OR:2.00; 95% CI:1.26–3.16) (Table 5). Thus, adolescents of low economic status were twice as likely to adhere to the “Diverse” pattern when compared to the “Mixed” dietary pattern. No significant associations were found between economic strata and the DPs, “Low consumption” and “Prudent”. No association was found between food insecurity and dietary patterns.

## 4. Discussion

Through the analysis of latent classes, four dietary patterns (Mixed, Low Consumption, Prudent and Diverse) were identified among the adolescents in this study. When evaluating the association between socioeconomic factors and dietary patterns, adolescents belonging to the lowest economic stratum were more likely to adhere to a “Diverse” DP. There was no association between food insecurity and identified dietary patterns.

In general, the literature points to the identification of two to five dietary patterns among adolescents, with an emphasis on three groups. One group was represented by the consumption of healthy or protective foods, such as fruits, vegetables, whole grains, fish, and legumes; another group was identified by foods with more industrialized or non-protective characteristics, such as processed cheeses, dairy products, sugar-sweetened beverages, fast food, sweets and desserts, animal products, refined cereals, fried foods, and snacks; and a third group was identified by the characteristics of a mixed or traditional diet in the place where the study was carried out [5,6,10,11,12,25,26,27]. Regarding the nomenclature of the DPs, there are discrepancies in the constitution of patterns with the same denomination, which may be due, in addition to the different methods and statistical approaches used, to cultural, socioeconomic, and geographic factors, not meaning that the DPs with the same name are composed of the same food items [6,8,25,27].

To determine dietary patterns that are increasingly representative of the studied population, the LCA has been used in developed countries [16,28] because its approach is useful for classifying individuals into exclusive classes based on the similarity of behaviors allowing, for example, interventions to modify eating practices that interfere with the health of individuals [8,13]. However, few studies have used LCA to identify dietary patterns among adolescents [11,12,15] and, given this scenario, the age range of the participants was also used to compare the results of this study with those reported in the literature.

Studies have identified that food items similar to those found in the “Mixed” DP were distributed in two distinct patterns called “traditional” and “bread and coffee/snack” [4,5,6,10,26,27,29,30]. In the present study, there was a greater probability of consuming (≥P75) milk, traditional Brazilian foods such as rice and beans, bakery products, and dairy products. Considering the adolescents classified in this dietary pattern, it was found that the majority were in the highest economic stratum “C and D”. Higher income has been associated with healthy dietary patterns, as it allows better access and availability of a variety of foods with better nutritional value [31]. 

The DP, “Low consumption”, formed by the majority of participants in this study, resembled the pattern called “Least diverse” identified by Thorne-Lyman et al. (2020) and is by low consumption of all food items, except potatoes [11]. The “Prudent” DP identified the consumption of fruits, vegetables, roots and tubers, flour and cereals, and poultry and fish, very similar to the DP termed “Healthy” identified by Borges et al. (2018) [5]. The DP, “Diverse”, of the present study presents contemporary elements of consumption with the intake of processed and ultra-processed foods, but also fresh and protective foods. This duality of eating behaviors was also found in the study by Thorne-Lyman et al. (2020) in the “More diverse” dietary pattern and indicates the eating paths that adolescents go through [11]. 

Several factors, including socioeconomic ones, influence human nutrition. A systematic review by Hinnig et al. (2018) pointed out that in developing countries, such as Brazil, the relationship between the consumption of unhealthy foods and socioeconomic level is not well established, finding studies in which unhealthy DP was associated with both populations with high and low economic levels [28]. The results of the association analyses of the present study identified that among adolescents from the lowest economic strata, there was a greater tendency towards consuming “Diverse” PA, composed of groups of ultra-processed foods, with high energy density, sugars and fats, but also of groups of fresh and healthy foods, such as fruits, roots and tubers, poultry and fish.

Longitudinal and cross-sectional studies have shown that DP made up of unhealthy food items was inversely associated with income and that this association may be a result of the high cost of healthy foods [28]. In addition, individuals with lower socioeconomic status are more likely to live in disordered and vulnerable environments, favoring access and consumption of unhealthy foods [32]. When studying Portuguese and Brazilian adolescents, Borges et al. (2018) found that the Western DP found in the two populations, geographically and socioeconomically distinct, is the result of global trends common to all social classes owing to the modification of the traditional/healthy DP to the Western/unhealthy DP [25].

It is crucial to understand the existing relationships between socioeconomic factors and dietary patterns in adolescence, as this transition period between childhood and adulthood has a great influence on the maintenance of healthy eating habits [25]. Socioeconomic status is also an important determinant of health, as it influences dietary patterns that comprise modifiable risk factors for various diseases, such as obesity, cardiovascular diseases and other non-transmissible chronic diseases that have been associated with the consumption of a “western” dietary pattern or “unhealthy” whose composition is similar to the “Diverse” DP of the present study [6,26,29,32].

With regard to FI at home, the present study did not demonstrate any association between FI and the identified dietary patterns. In contrast, previous studies have observed lower adherence to DP composed of animal protein, fruits, vegetables, cereals, and dairy products among adolescents with food insecurity. However, they also did not find an association between adherence to the “unhealthy/western” pattern and FI [7,33]. Individuals with household food insecurity tend to make worse dietary choices and favor unhealthy foods with low nutrient content and high energy density, in addition to inadequate consumption of fruits and vegetables, animal proteins, whole grains, and dairy products. FI acts as a psychological stressor inducing behavioral effects that interfere with the long-term health of children and adolescents [7,33,34].

This study has some limitations: (i) data on food consumption were obtained through a single self-reported instrument, the FFQ, which has the potential for recall and social desirability bias. However, we used a validated FFQ in the study age group. (ii) The researchers determined the criteria for clustering food items for the subsequent LCA. However, we chose to consider both the extent and purpose of processing food items and the similarity in nutrient content. (iii) We did not assess the potential influence of other unmeasured variables, such as culture, nutrition, access to food, conformation of the environment around the household, availability of food at school, and peer influence. (iv) This was a cross-sectional study; therefore, causality could not be inferred. 

The strengths of the study are as follows: (i) The use of data from a representative and probabilistic sample of the age group of students from state public schools allowed a robust analysis of food intake. (ii) The identification of dietary patterns using a precise, sophisticated, person-oriented statistical technique that allows the classification of individuals into distinct classes based on similarities in eating behavior. (iii) The nomenclature of the dietary patterns was the food items with the highest probability of item response and based on the scientific literature on the subject.

## 5. Conclusions

Four dietary patterns were identified among adolescents in this study. The “Mixed” DP was marked by greater adherence to food groups such as milk, oils and fats, bakery products, rice and pasta and vegetables. Approximately half of the adolescents adhered to the “Low consumption” DP, with a low probability of consuming practically all food groups (≥P75). The highest probability of consumption (≥P75) of fruits, vegetables, roots and tubers, flour and cereals was identified in the PD termed “Prudent”. The fourth PD, “Diverse”, was characterized by a high probability of consumption of practically all food groups in the ≥P75 percentile level. The lowest economic stratum was positively associated with the “Diverse” dietary pattern, highlighting the need to link preventive measures, policies, or intervention strategies aimed at improving the quality of the diet and access to food for adolescents. No association was found between household food insecurity and dietary patterns. Therefore, promoting a healthy diet for this age group should be a priority for disease prevention and control at all socioeconomic levels.

## Figures and Tables

**Table 1:** Composition of food groups.

Food Groups	Composition
Vegetables	Lettuce, kale, cabbage, pumpkin, carrot, tomato, chayote, gherkin, beetroot, okra, vegetable salad.
Fruits	Pineapple, avocado, acerola, banana, plantain, cashew, jackfruit, papaya, mango, apple, watermelon, melon, orange, tangerine, strawberry, açaí, and natural fruit juice or pulp.
Roots and tubers	Cassava, yam, potato, and sweet potato.
Red meat	Beef, viscera, and salted meat.
Poultry and fish	Chicken, eggs, fish, and seafood.
Milks	Whole and skimmed milk (powder or liquid).
Rice and noodles	Rice and noodles (white or brown), and noodle soup.
Legumes	Beans and peanuts.
Typical foods	Acarajé, feijoada, and tropeiro beans.
Flours and cereals	Cassava and corn flour, dairy flour, corn flakes (couscous), oat flakes, popcorn, and granola.
Bakery products	White or wholemeal bread, cakes, savory biscuits, and sweet biscuits.
Dairy products	Fermented milk, yogurt, cream cheese, and cheese.
Sweets and sugars	Candy, gum, lollipop, chocolate bar, ice cream, yogurt ice cream, gelatin, guava paste, marmalade, homemade sweets, powdered chocolate, ready-to-drink chocolate, and added sugar.
Oils and fats	Vegetable oils, olive oil, palm oil, butter, and margarine.
Sugar-sweetened beverages	Traditional and diet/light/zero-sugar sodas, soft drinks and artificial juices, energy drinks, and flavored carbonated waters.
Fast food	Fried snacks, packaged snacks, hot dogs, snacks/hamburgers, French fries or straws, frozen pizza and lasagna, ready-to-eat soups, instant noodles, and sausages.

**Table 2:** Statistical criteria for selection of the model of latent class of adolescent dietary patterns.

No. Latent Class	No. Free Parameters	AIC	BIC	BIC-Adjusted	Entropy	LMR-LRT	BS-LRT *p*-Value *p*/k–1
Model 1							
2 LC	33	21,895.270	22,063.652	21,958.831	0.864	−12,407.001; *p* < 0.0000	−12,407.001; *p* < 0.0000
3 LC	50	21,533.777	21,788.902	21,630.082	0.767	−10,914.635; *p* < 0.0000	−10,914.635; *p* < 0.0000
4 LC	67	21,453.861	21,795.728	21,582.909	0.714	−10,716.889; *p* < 0.3987	−10,716.889; *p* < 0.0000
5 LC	84	21,399.056	21,827.666	21,560.848	0.722	−10,659.930; *p* < 0.4285	−10,659.930; *p* < 0.0000
Model 2							
2 LC	33	17,324.714	17,493.097	17,388.275	0.866	−9858.671; *p* < 0.0000	−9858.671; *p* < 0.0000
3 LC	50	16,998.134	17,253.259	17,094.438	0.780	−8629.357; *p* < 0.0000	−8629.357; *p* < 0.0000
4 LC	67	16,937.451	17,279.318	17,066.499	0.754	−8449.067; *p* < 0.1078	−8449.067; *p* < 0.0000
5 LC	84	16,918.858	17,347.468	17,080.650	0.700	−8401.725; *p* < 0.5316	−8401.725; *p* < 0.0128
Model 3							
2 LC	33	19,277.226	19,445.609	19,340.787	0.870	−10,983.494; *p* < 0.0000	−10,983.494; *p* < 0.0000
3 LC	50	18,960.449	19,215.574	19,056.754	0.770	−9605.613; *p* < 0.0000	−9605.613; *p* < 0.0000
4 LC	67	18,904.756	19,246.623	19,033.804	0.737	−9430.225; *p* < 0.5203	−9430.225; *p* < 0.0000
5 LC	84	18,849.017	19,277.627	19,010.809	0.722	−9380.809; *p* < 0.2804	−9380.809; *p* < 0.0000

AIC, Akaike Information Criterion; BIC, Bayesian Information Criterion; LMR-LRT, Vuong–Lo–Mendell–Rubin Likelihood Ratio Test; BS-LRT Bootstrap Likelihood Ratio Test. Model 1: Consumption of food groups in tertiles. Model 2: Consumption of food groups in quintiles. Model 3: Consumption of food groups in percentiles. LC, latent class.

**Table 3:** Conditional probabilities of food consumption considering four dietary patterns, derived from latent class analysis, in adolescents.

Food Groups	Dietary Patterns			
	Mixed	Low Consumption	Prudent	Diverse
Vegetables	26.6	7.6	**45.0**	59.9
Fruits	17.9	3.1	**49.8**	**80.8**
Roots and tubers	23.2	5.8	**44.7**	**70.5**
Red meat	27.9	6.7	**35.4**	**75.5**
Poultry and fish	27.0	6.6	**38.2**	**70.5**
Milks	**69.6**	**23.6**	20.6	59.8
Rice and noodles	**32.3**	6.7	33.8	**70.9**
Legumes	**31.8**	7.9	26.8	47.3
Typical foods	25.8	10.5	31.4	67.8
Flours and cereals	16.7	9.0	**42.0**	**70.7**
Bakery products	**39.9**	5.8	23.1	**76.9**
Dairy products	**36.3**	4.9	32.4	**71.5**
Sweets and sugars	**34.0**	4.4	24.2	**87.8**
Oils and fats	**61.9**	9.3	9.6	54.6
Sugar-sweetened beverages	25.3	7.8	33.3	**74.1**
Fast food	**34.7**	4.3	29.4	**81.1**
Mean posterior probability (unconditional)	77.7	90.0	76.0	90.7

The highest probabilities defined dominant food groups for each cluster and are shown in bold.

**Table 4:** Prevalence of dietary patterns among adolescents according to socioeconomic factors and food insecurity.

Variables	All Participants (*n* = 1215)		Dietary Patterns								*p* Value *
			Mixed (*n* = 192)		Low Consumption (*n* = 644)		Prudent (*n* = 209)		Diverse (*n* = 170)		
	n	%	n	%	n	%	n	%	n	%	
Sex											0.199
Male	513	42.2	72	14.0	272	53.0	100	19.5	69	13.5	
Female	702	57.8	120	17.1	372	53.0	109	15.5	101	14.4	
Age											0.939
<14 years	528	43.5	84	15.9	281	53.2	87	16.5	76	14.4	
≥14 years	687	56.5	108	15.7	363	52.8	122	17.8	94	13.7	
Economic strata											0.022 **
Stratum C e D	619	51.0	106	17.1	339	54.8	105	17.0	69	11.1	
Stratum E	594	49.0	86	14.5	303	51.0	104	17.5	101	17.0	
BFIS											0.766
Food Security	410	33.7	61	14.9	230	56.1	68	16.6	51	12.4	
FI mild	461	37.9	80	17.4	228	49.5	85	18.4	68	14.8	
FI moderate	251	20.7	40	15.9	134	53.4	41	16.3	36	14.3	
FI severe	93	7.7	11	11.8	52	55.9	15	16.1	15	16.1	

BFIS: Brazilian Scale of Food Insecurity. FI: food insecurity. * Pearson’s chi square test. ** *p*< 0.05.

**Table 5:** Association between dietary patterns and socioeconomic factors and food insecurity in a sample of adolescents.

Variables	Dietary Patterns					
	Low Consumption		Prudent		Diverse	
	OR _Bivariate_ (95% CI)	OR _Multivariate_ (95% CI)	OR _Bivariate_ (95% CI)	OR _Multivariate_ (95% CI)	OR _Bivariate_ (95% CI)	OR _Multivariate_ (95% CI)
Sex						
Male	Ref.	Ref.	Ref.	Ref.	Ref.	Ref.
Female	0.90 (0.62–1.30)	0.88 (0.60–1.28)	0.70 (0.44–1.10)	0.70 (0.44–1.11)	0.79 (0.50–1.27)	0.78 (0.48–1.24)
Age						
<14 years	Ref.	Ref.	Ref.	Ref.	Ref.	Ref.
≥14 years	1.12 (0.78–1.62)	1.11 (0.77–1.59)	1.20 (0.77–1.86)	1.15 (0.73–1.80)	0.98 (0.62–1.55)	0.93 (0.58–1.47)
Economics strata						
Strata C e D	Ref.	Ref.	Ref.	Ref.	Ref.	Ref.
Strata E	1.33 (0.93–1.92)	1.38 (0.94–2.03)	1.40 (0.90–2.20)	1.39 (0.88–2.20)	2.00 (1.26–3.16)	2.02 (1.26–3.24)
BFIS						
Food Security	Ref.	Ref.	Ref.	Ref.	Ref.	Ref.
FI mild	0.70 (0.48–1.01)	0.71 (0.49–1.03)	1.0 (0.64–1.60)	1.01 (0.63–1.61)	0.94 (0.59–1.50)	0.93 (0.58–1.49)
FI moderate	1.08 (0.69–1.67)	1.02 (0.65–1.61)	1.05 (0.60–1.83)	0.99 (0.56–1.75)	1.10 (0.64–1.91)	0.95 (0.55–1.66)
FI severe	1.65 (0.77–3.50)	1.60 (0.74–3.46)	1.16 (0.47–2.85)	1.10 (0.45–2.71)	1.53 (0.64–3.63)	1.39 (0.58–3.33)

BFIS: Brazilian Scale of Food Insecurity. FI: food insecurity. Adjusted for all other variables in the table.

## Data Availability

All data generated or analyzed during this study are available from the corresponding author upon reasonable request.

## Supplementary Materials

The following supporting information can be downloaded at: https://www.mdpi.com/article/10.3390/nu15204344/s1, Table S1: Cutoff points, in grams of 75th percentile intake of food groups according to dietary pattern.
