# Supplementary material for: Association between Socioeconomic Factors, Food Insecurity, and Dietary Patterns of Adolescents: A Latent Class Analysis
Source: Nutrients. 2023 Oct 12;15(20):4344. doi: 10.3390/nu15204344 (PMC10610405; doi:10.3390/nu15204344)
Supplement: Supplementary file 1 [file nutrients-15-04344-s001.zip › nutrients-2630214-supplementary.pdf]

Table S1. Cutoff points, in grams of 75th percentile intake of food groups according to dietary pattern.

Food Groups (grams)	All participants (n=1,215)	Dietary Patterns							
		Mixed (n=192)		Low Consumption (n=644)		Prudent (n=209)		Diverse (n=170)	
		0	1	0	1	0	1	0	1
	Means (SE)	Means (SE)	Means (SE)	Means (SE)	Means (SE)	Means (SE)	Means (SE)	Means (SE)	Means (SE)
Vegetables	117.9 (5.1)	57.7 (4.2)	292.0 (16.1)	48.9 (2.0)	221.6 (7.1)	66.4 (5.4)	294.6 (13.3)	75.6 (6.1)	371.3 (25.6)
Fruits	716.4 (24.9)	485.5 (19.7)	1,450.9 (101.3)	318.5 (7.6)	1,504.9 (111.0)	527.7 (29.8)	1,517.0 (40.8)	592.8 (52.8)	2,002.3 (73.6)
Roots and tubers	60.6 (3.4)	21.0 (1.4)	135.7 (10.9)	18.4 (0.9)	146.4 (13.0)	25.6 (2.1)	150.2 (7.5)	23.0 (3.2)	238.9 (13.3)
Red meat	68.3 (2.5)	41.3 (2.2)	165.5 (11.0)	30.3 (1.1)	141.6 (8.1)	39.0 (2.2)	155.6 (7.6)	51.1 (4.0)	189.4 (7.4)
Poultry and fish	105.8 (5.2)	54.1 (3.2)	234.8 (17.8)	39.3 (1.0)	220.9 (17.4)	57.7 (2.6)	278.0 (20.6)	72.2 (5.1)	342.1 (18.8)
Milks	31.5 (1.2)	16.2 (1.9)	65.7 (2.2)	10.6 (0.5)	53.8 (1.2)	15.2 (1.2)	65.5 (4.2)	21.2 (1.6)	77.0 (3.0)
Rice and noodles	288.4 (9.0)	187.9 (11.2)	589.2 (23.1)	147.3 (5.0)	497.3 (12.3)	192.6 (9.9)	586.2 (24.9)	256.3 (15.0)	791.5 (34.3)
Legumes	181.7 (4.5)	146.1 (8.0)	368.8 (12.1)	111.8 (4.5)	380.7 (17.7)	149.6 (8.3)	363.1 (14.7)	171.2 (9.4)	413.5 (17.8)
Typical foods	186.1 (8.3)	90.4 (5.3)	424.6 (25.9)	74.4 (2.3)	410.4 (28.8)	91.5 (3.8)	473.2 (35.7)	129.1 (8.9)	590.4 (25.9)
Flours and cereals	70.3 (2.7)	31.2 (1.8)	194.9 (18.2)	23.6 (0.8)	146.8 (10.3)	36.3 (2.1)	171.7 (7.8)	47.0 (3.1)	243.6 (13.8)
Bakery products	237.0 (5.6)	191.5 (8.1)	462.5 (15.8)	130.9 (4.1)	437.0 (34.0)	179.9 (7.1)	458.3 (17.3)	248.7 (10.2)	543.6 (18.5)
Dairy products	111.2 (5.1)	42.2 (3.3)	285.9 (14.6)	33.2 (1.4)	226.8 (12.2)	48.9 (2.9)	304.1 (19.2)	60.8 (6.1)	405.6 (20.4)
Sweets and sugars	421.1 (14.0)	289.8 (10.9)	860.0 (44.3)	206.7 (5.6)	709.1 (25.0)	303.3 (8.4)	800.5 (32.7)	326.4 (27.6)	1,138.3 (66.5)
Oils and fats	29.2 (0.9)	23.2 (1.9)	63.0 (2.3)	15.8 (0.7)	58.2 (1.7)	20.4 (1.0)	54.4 (1.5)	24.1 (1.4)	66.9 (2.4)
Sugar-sweetened beverages	679.4 (22.7)	483.4 (13.8)	1,468.3 (75.0)	312.0 (8.4)	1,384.0 (66.3)	423.2 (20.8)	1,379.9 (43.8)	608.3 (31.2)	1,909.5 (78.0)
Fast food	299.1 (11.0)	188.0 (7.2)	646.3 (32.5)	129.8 (3.5)	544.7 (34.0)	190.2 (7.7)	615.7 (37.4)	241.6 (14.8)	889.3 (39.0)

0: <P75; 1: >P75. SE: Standard error.

The values in this table correspond to the values (means) in grams of the total food items that comprise each food group.
